# BUILDING AND SUSTAINING ORGANIZATIONAL PARTNERSHIPS TO SUPPORT DISASTER PREPAREDNESS IN OLDER ADULTS

**DOI:** 10.1093/geroni/igae098.1607

**Published:** 2024-12-31

**Authors:** Emily Killian, Lena Thompson, Miranda Fisher, Nicholas Ostrem, Tiffany Wunderlich, Brigid Smith, Sato Ashida

**Affiliations:** University of Iowa College of Public Health, Iowa City, Iowa, United States; University of Minnesota Duluth, Duluth, Minnesota, United States; University of Iowa, Iowa City, Iowa, United States; University of Iowa, Iowa City, Iowa, United States; University of Iowa, Iowa City, Iowa, United States; University of Iowa, Iowa City, Iowa, United States; University of Iowa, Iowa City, Iowa, United States

## Abstract

Long-term implementation of programs for community-based older adults requires collaboration among various organizations. Strengthening personal and community support systems is critical in supporting older adults before, during, and after disasters. The Disaster PrepWise (DPW) program was developed through community-engaged processes involving disaster management and public health agencies as well as aging service providers to ensure the usability and acceptability of the program. DPW implementation infrastructure and protocols were subsequently developed using the concepts of the Practical, Robust Implementation, and Sustainability Model (PRISM) framework. Organizational partners participated in periodic Stakeholder Advisory Board (SAB) meetings and collaboratively developed a program delivery infrastructure. The developed infrastructure was pilot-tested and evaluated by conducting interviews from July to September 2023 with various stakeholders: program participants (n=18), SAB members (n=6), and interventionists (n=7). Strong alignment between the DPW program and their values and the opportunity to work with “like-minded” people motivated organizations to participate. Organizations involved since the program development stage, over 10 years ago, were especially committed to supporting the development of implementation infrastructure. SAB members highlighted additional benefits to older adults as this infrastructure can connect DPW participants to other social services in their community through cross-sectorial collaborations. Participants identified barriers to long-term sustainability such as a lack of sustained resources, identifying and training interventionists, staff turnover, and difficulty expanding to reach partners in rural areas. These collective challenges underscore the complexity of maintaining such initiatives and the necessity for comprehensive strategies to sustain them.

